# Antiretroviral Treatment Outcomes amongst Older Adults in a Large Multicentre Cohort in South Africa

**DOI:** 10.1371/journal.pone.0100273

**Published:** 2014-06-20

**Authors:** Geoffrey Fatti, Eula Mothibi, Graeme Meintjes, Ashraf Grimwood

**Affiliations:** 1 Kheth’Impilo, Cape Town, South Africa; 2 Division of Infectious Diseases and HIV Medicine, Department of Medicine, University of Cape Town, Cape Town, South Africa; 3 Institute of Infectious Disease and Molecular Medicine, University of Cape Town, Cape Town, South Africa; 4 Department of Medicine, Imperial College, London, United Kingdom; University of Washington, United States of America

## Abstract

**Introduction:**

Increasing numbers of patients are starting antiretroviral treatment (ART) at advanced age or reaching advanced age while on ART. We compared baseline characteristics and ART outcomes of older adults (aged ≥55 years) vs. younger adults (aged 25–54 years) in routine care settings in South Africa.

**Methods:**

A multicentre cohort study of ART-naïve adults starting ART at 89 public sector facilities was conducted. Mortality, loss to follow-up (LTFU), immunological and virological outcomes until five years of ART were compared using competing-risks regression, generalised estimating equations and mixed-effects models.

**Results:**

4065 older adults and 86,006 younger adults were included. There were more men amongst older adults; 44.7% vs. 33.4%; RR = 1.34 (95% CI: 1.29–1.39). Mortality after starting ART was substantially higher amongst older adults, adjusted sub-hazard ratio (asHR) = 1.44 over 5 years (95% CI: 1.26–1.64), particularly for the period 7–60 months of treatment, asHR = 1.73 (95% CI: 1.44–2.10). LTFU was lower in older adults, asHR = 0.87 (95% CI: 0.78–0.97). Achievement of virological suppression was greater in older adults, adjusted odds ratio = 1.42 (95% CI: 1.23–1.64). The probabilities of viral rebound and confirmed virological failure were both lower in older adults, adjusted hazard ratios = 0.69 (95% CI: 0.56–0.85) and 0.64 (95% CI: 0.47–0.89), respectively. The rate of CD4 cell recovery (amongst patients with continuous viral suppression) was 25 cells/6 months of ART (95% CI: 17.3–33.2) lower in older adults.

**Conclusions:**

Although older adults had better virological outcomes and reduced LTFU, their higher mortality and slower immunological recovery warrant consideration of age-specific ART initiation criteria and management strategies.

## Introduction

Increasing numbers of people are reaching advanced ages while receiving antiretroviral treatment (ART) in sub-Saharan Africa, as ART coverage has rapidly expanded and survival and life expectancy have increased due to ART [1]. Older adults living with HIV numbered over three million people in sub-Saharan Africa in 2011 [2]. This figure is set to triple to over 9 million people by 2040, with older adults accounting for 27% of the HIV-infected population as the proportion of young adults declines by one fourth [2].

Multiple comorbidities, polypharmacy, accelerated biological aging and cognitive decline all potentially complicate HIV treatment in older adults [3]–[6]. Polypharmacy in patients receiving ART increases the risks of potentially serious drug-drug interactions, potentially leading to drug toxicity, and may contribute to inadequate adherence resulting in viral breakthrough and viral resistance [7]. Adherence to ART has been found to be generally better amongst older age groups than younger age groups; however, adherence is reduced in the presence of cognitive impairment resulting in poorer treatment response [5], [7]. ART outcomes amongst older adults have been extensively investigated in small cohorts in developed countries [3], [8]–[15]. In contrast, there are limited data of the effectiveness of ART in older adults in resource-limited settings, where over 90% HIV-infected people live [16]. The studies that have been performed have shown inconsistent results regarding both mortality and loss to follow-up when comparing patients in older age to younger patients receiving ART [17]–[19]. This study compared baseline characteristics and clinical, virologic and immunologic outcomes of older adults with those of younger adults receiving ART from 89 public sector facilities in four South African provinces.

## Methods

### Ethics Statement

The study was approved by the University of Cape Town Human Research Ethics Committee. As all analysed data were routinely collected for all ART patients, patient informed consent was not needed for clinical records to be used in the study, as per the approved protocol. Patient records were anonymized and de-identified prior to analysis.

### Study Design and Setting

A multicentre cohort study utilising routine clinical data was conducted at ART facilities supported by Kheth’Impilo, a non-governmental organisation that supports the South African Department of Health. In South Africa, 1.8 million people were receiving ART by mid-2011, with adult ART coverage of those eligible being 52% [20]. Kheth’Impilo provides clinical staff, infrastructure, health system strengthening initiatives and technical assistance as well as manages a clinic-linked community-based adherence-support program for ART patients [21]–[23]. Facilities were located in both urban and rural areas of four provinces (Western Cape, Eastern Cape, KwaZulu-Natal, and Mpumalanga) with provincial antenatal HIV prevalences ranging between 18% and 37% [24]. Seventeen facilities were located at district or regional hospitals, with the remainder located at primary healthcare facilities.

### Inclusion Criteria, Outcomes and Definitions

Adults with CD4 cell counts ≤200 cells/µl and/or a WHO stage IV defining illness were eligible to start ART as per the 2004 South African national treatment guidelines. [25] From April 2010, ART eligibility criteria were expanded to include adults with CD4 cell counts ≤350 cells/µl if they were pregnant or diagnosed with active tuberculosis [26]. Standardised first-line regimens consisted of two nucleoside reverse transcriptase inhibitors and one non-nucleoside reverse transcriptase inhibitor.

All adults (≥25 years of age) not previously enrolled for ART starting triple-drug combination ART between January 1^st^, 2004 and September 30^th^, 2010 with documented date of birth, gender and date of starting ART were included in analyses. The WHO does not have a standard age definition of an older adult, but suggests a cutoff of either ages 50 or 55 years and over to define an older person in Africa [27]. We defined older adults as those starting ART aged 55 years and over, as has previously been used in Africa [28]. Patients were followed up from the start of ART until the earliest of last clinic follow-up visit (for patients dying, transferring out or lost to follow-up [LTFU]), five years from starting ART, NGO exit from a site (7 sites), or March 31^st^, 2011.

Outcome measures were: time to all cause-mortality after starting ART, time to LTFU, proportions of patients achieving virological suppression on ART, time until virological rebound (since initial virological suppression), time until confirmed virological failure after starting ART, proportions of patients switching to second-line ART during the study period and changes in CD4 cell counts from baseline. Additionally, CD4 cell count changes were analysed in a subset of patients who had continuous viral suppression on ART. Deaths were ascertained by health care workers at facilities, or through the report of a Kheth’Impilo community-based adherence worker. A patient was defined as LTFU if no visits to the clinic occurred for 187 days or more [29], (180 days +7 days for potential data capturing backlogs). Patients who missed appointments would initially be traced by telephone, and where capacity was available and if prior consent was obtained, a community adherence worker or district tracing team would perform a home visit. Virological suppression was defined as a viral load <400 copies/ml. Viral rebound was defined as a viral load >400 copies/ml after having achieved a suppressed viral load during the first 12 months of treatment. Confirmed virological failure was defined as two consecutive viral loads >1000 copies/ml as per national guidelines [30]. CD4 cell count was measured at ART initiation and at six-monthly intervals, and viral load was measured six-monthly on treatment. Baseline viral load measurements were available for a small minority of patients, and thus not included in analyses. Laboratory measurements were performed by the South African National Health Laboratory Service.

### Data Collection and Statistical Analyses

Individual-level patient data were collected prospectively for patient monitoring purposes by designated site-based data capturers at each patient visit using standardised custom-designed electronic databases, which were regularly pooled to a central data warehouse using standard operating procedures. Regular data cleaning and quality control procedures were implemented.

Characteristics at the start of ART of older and younger adults were compared using Wilcoxon’s Rank-Sum and Pearson’s χ^2^ tests for continuous and categorical data, respectively. Cumulative incidence functions and competing-risks regression using the method of Fine and Gray were used to estimate crude and adjusted measures of time until mortality and LTFU after starting ART [31]. Competing-risks analysis accounts for the fact that two event types (death and LTFU) play a role in failure and these events are not independent, i.e. one event occurring precludes or alters the probability of the occurrence of the other event [32]. This is an appropriate method to model LTFU and mortality in ART programs in low-income settings [33]. Kaplan-Meier estimates, the logrank test and Cox proportional hazards regression were used to analyse time until viral rebound (from initial viral suppression) and time until confirmed virological failure from starting ART. Multivariable population-averaged generalised estimating equations with robust variance estimates were used to analyse factors associated with viral suppression. Log-binomial regression was used to analyse proportions who switched to second-line treatment. Mixed-effects linear models were used to analyse factors associated with changes in CD4 cell counts after starting ART [34], [35].

The following *a priori* specified covariates that were plausible confounders were eligible to be included in multivariable regression models in order to control for confounding: patient-related variables- gender, baseline CD4 cell count, baseline World Health Organisation (WHO) clinical stage, baseline tuberculosis treatment, year of starting ART, pregnancy when starting ART, initial ART regimen, receipt of community adherence worker support, time on ART; site-related variables- province, urban/rural site, hospital/primary healthcare facility. Covariates were included in multivariable models where their inclusion produced a ≥10% shift in the point estimate of the prime exposure variable (age category) [36]. Analyses were performed with Stata version 11.1 (College Station, TX, USA).

## Results

Database records for 136,524 patients were screened for inclusion in the study. The following were excluded: 5271 from four sites that did not collect baseline demographic or outcome data; 22,096 who were transferred-in to sites already receiving ART and 19,086 who were aged <25 years when starting ART. A total of 90,071 patients were thus included, of whom 4065 (4.5%) were aged ≥55 years when starting ART and 86,006 (95.5%) were aged <55 years.

Characteristics at the start of ART (baseline) are shown in table 1. The median ages of older and younger patients were 58.6 years (IQR: 56.6–62.2) and 35.2 years (IQR: 30.6–41.1), respectively. 590 (14.5%) older adults were age ≥65 years when starting ART. There was a higher proportion of men amongst older adults (44.7% vs. 33.4%; P<0.0001). Older patients had a lower proportion who had extreme immunodeficiency (CD4 cell count <50 cells/µL) at baseline (14.9% vs. 19.6%; P<0.0001) Older patients had a slightly lower proportion with advanced (stage IV) World Health Organization clinical stage disease (10.6% vs. 12.0%; P<0.0001) as well as a lower proportion who were receiving tuberculosis treatment at baseline (11.4% vs. 13.0%; P<0.0001). A higher proportion of older adults were treated at rural facilities (28.7% vs. 19.3%; P<0.0001).

**Table 1:** Characteristics of older and younger adults starting antiretroviral treatment in South Africa.

	Older adults(ages ≥55 years)	Younger adults(ages 25–54 years)	P-value
**Participants included, n (%);** (N = 90,071)	4065 (4.5%)	86,006 (95.5%)	
**Median age, years (IQR);** (N = 90,071)	58.6 (56.6–62.2)	35.2 (30.6–41.1)	
**Male gender;** (N = 90,071)	1818 (44.7)	28,751 (33.4)	<0.0001
**Median CD4 cell count, cells/µL (IQR);** (N = 72,685)	124 (64–176)	131 (76–178)	<0.0001
**CD4 cell count categories, n (%)**			<0.0001
<50 cells/µL	496 (14.9)	13,610 (19.6)	
50–100 cells/µL	717 (21.5)	14,153 (20.4)	
101–200 cells/µL	1707 (51.3)	32,727 (47.2)	
>200 cells/µL	410 (12.3)	8,865 (12.8)	
**WHO clinical staging, n (%);** (N = 58,611)			<0.0001
Stage I	123 (4.7)	3504 (6.3)	
Stage II	538 (20.5)	12,003 (21.4)	
Stage III	1683 (64.2)	33,751 (60.3)	
Stage IV	278 (10.6)	6731 (12.0)	
**Tuberculosis treatment, n (%);** (N = 74,153)	407 (11.4)	9181 (13.0)	<0.0001
**Pregnancy, n (%);** (N = 80,027)	0 (0)	3142 (4.1)	<0.0001
**Received community-based adherence support,** **n (%);** (N = 90,071)	1295 (31.9)	23,283 (27.1)	<0.0001
**Initial regimen ** ***nucleoside*** **reverse-transcriptase** **inhibitor, n (%);** (N = 70,589)^1^			<0.0001
stavudine	2712 (79.9)	52,738 (78.5)	
zidovudine	115 (3.4)	1822 (2.7)	
tenofovir	569 (16.8)	12,633 (18.8)	
**Efavirenz-based initial regimen, n (%);** (N = 70,773)^2^	3211 (94.3)	49,599 (73.6)	<0.0001
**Year of starting ART, median (IQR)**; (N = 90,071)	2009 (2008–2010)	2009 (2007–2010)	<0.0001
**Rural treatment facility, n (%);** (N = 90,071)	1168 (28.7)	16,569 (19.3)	<0.0001
**Hospital based treatment facility, n (%);** (N = 90,071)^3^	1176 (28.9)	22,354 (26.0)	<0.0001

1 all patients additionally received lamivudine in initial regimen.

2 patients received nevirapine when not receiving efavirenz in initial regimen.

3 all other patients were managed at primary healthcare facilities.

ART-antiretroviral treatment; WHO-World Health Organization; IQR- interquartile range.

**Figure 1:**
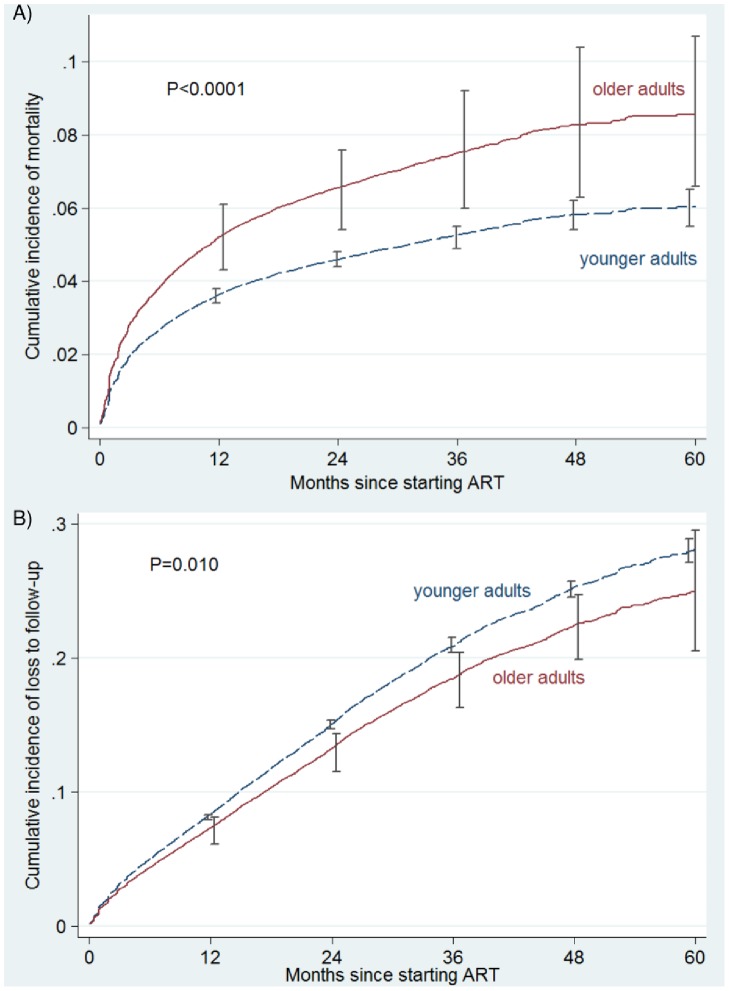
Covariate-adjusted competing risks cumulative incidence curves of A) mortality; B) loss to follow-up after starting antiretroviral treatment amongst older and younger adults in South Africa. Error bars are 95% confidence intervals.

The total observation time was 121,021 person-years, with median follow-up durations of 12.0 months (IQR: 4.7–22.7 months) and 13.1 months (IQR: 5.5–25.0 months) amongst older and younger adults, respectively. During the study period, 344 (8.5%) older adults and 5002 (5.8%) younger adults were recorded as having died. A total of 488 (12.0%) older adults and 11,082 (12.9%) younger adults became LTFU. The competing-risks cumulative incidence estimate of mortality was substantially higher amongst older adults, with crude cumulative incidences of 13.1% (95% CI: 11.0%–15.3%) and 8.2% (95% CI: 7.8%−8.6%) in older and younger adults after 5 years of ART, respectively. The covariate-adjusted competing-risks cumulative incidence estimates of mortality are illustrated in figure 1A. The adjusted sub-hazard ratio (asHR) of mortality in older vs. younger adults over 5 years was 1.44 (95% CI: 1.26–1.64). Covariates included in the model were as follows: male gender, asHR = 1.40 (95% CI: 1.30–1.49); baseline WHO clinical stages 3 & 4 (compared to stages 1 & 2), asHR = 1.45 (95% CI: 1.31–1.61); baseline CD4 cell count, asHR = 0.99 (95% CI: 0.99–0.99); pregnancy at baseline, asHR = 0.55 (95% CI: 0.36–0.83); year of starting ART, asHR = 0.81 (95% CI: 0.78–0.83); receipt of community adherence-support worker, asHR = 0.88 (95% CI: 0.80–0.97) and rural facility, asHR = 2.02 (95% CI: 1.79–2.28).

During the earlier ART period (months 0–6 of treatment), mortality rates for older and younger adults were 100 and 73 deaths/1000 person-years, respectively; and mortality rates during months 7–60 (later ART period) were 36 and 18 deaths/1000 person-years for older and younger adults, respectively. During the early and later ART periods the asHRs (for older vs. younger adults) were 1.34 (95% CI: 1.14–1.57) and 1.73 (95% CI: 1.44–2.10), respectively. The absolute increase in mortality amongst older adults was thus greater during the earlier period (2.7 deaths/100 person-years); however, the relative increase reflected in the asHRs was greatest during the later ART period.

Mortality rates for older adults during the later ART periods by age categories were as follows: ages 55–59 years = 34 deaths/1000 person-years; ages 60–64 years = 37 deaths/1000 person-years and ages 65–69 years = 47 deaths/1000 person-years. (For comparison, age-specific mortality rates amongst the older South African general population in 2010 were substantially lower: ages 55–59 years = 21 deaths/1000 person-years; ages 60–64 years = 27 deaths/1000 person-years and ages 65–69 years = 31 deaths/1000 person-years) [37].

The crude cumulative incidences of LTFU were 22.9% (95% CI: 19.8%–26.0%) and 25.2% (95% CI: 24.5%–25.9%) after 5 years of ART amongst older and younger adults, respectively. In adjusted analyses, LTFU was lower amongst older adults asHR = 0.87 (95% CI: 0.78–0.97), as illustrated in Figure 1B. Covariates included in the model of LTFU were as follows: male gender, asHR = 1.22 (95% CI: 1.16–1.28); baseline WHO clinical stages III & IV, asHR = 1.13 (95% CI: 1.06–1.20); baseline CD4 cell count, asHR = 0.99 (95% CI: 0.99–1.00); pregnancy at baseline, asHR = 1.23 (95% CI: 1.09–1.40); year of starting ART, asHR = 1.20 (95% CI: 1.18–1.22); receipt of community adherence-support worker, asHR = 0.69 (95% CI: 0.65–0.74) and rural facility, asHR = 0.77 (95% CI: 0.69–0.87).

**Figure 2:**
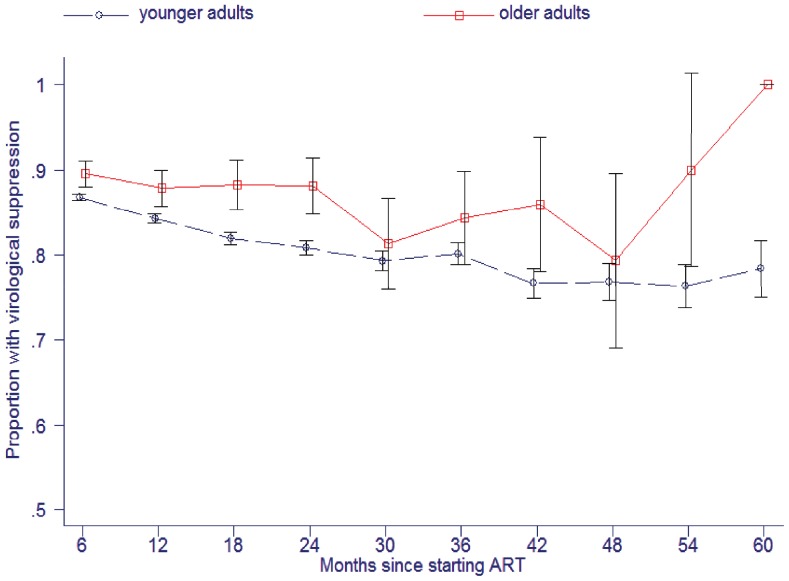
Proportions of older and younger adults achieving viral suppression according to duration of antiretroviral treatment in South Africa. Error bars are 95% confidence intervals.

The proportion of patients achieving viral suppression on ART was greater in older adults, being 88.0% (95% CI: 86.9%–89.0%) vs. 83.7% (95% CI: 83.4%–83.9%) at any time-point on treatment (*P*<0.0005; n = 88,467 observations). Figure 2 shows higher viral suppression amongst older adults at each six monthly interval on treatment. Modelled over five years of treatment, the adjusted odds ratio (aOR) for viral suppression in older vs. younger adults was 1.42 (95% CI: 1.23–1.64). Covariates included in the model were: male gender, aOR = 0.86 (95% CI: 0.83–0.90); year of starting ART, aOR = 0.87 (95% CI: 0.86–0.88) and months since starting ART, aOR = 0.98 (95% CI: 0.98–0.98).

**Figure 3:**
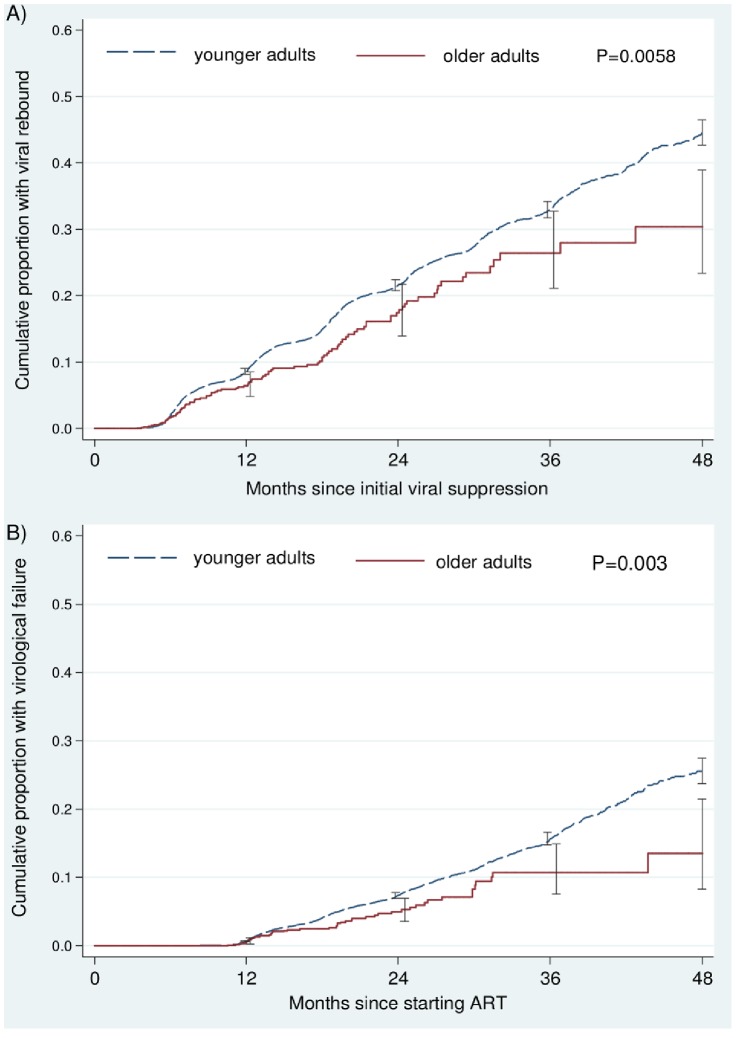
Cumulative probabilities of A) viral rebound after initial suppression and B) confirmed virological failure amongst older and younger adults after starting antiretroviral treatment in South Africa. Error bars are 95% confidence intervals.

Figure 3A shows cumulative probabilities of viral rebound following initial viral suppression. Older adults had lower probabilities of rebound, being 26.4% (95% CI: 21.1%–32.8%) in older adults and 33.0% (95% CI: 31.7%–34.3%) in younger adults three years after initial suppression. (*P* = 0.0058). After adjustment, older adults had a 31% lower risk of viral rebound, adjusted hazard ratio (aHR) = 0.69 (95% CI: 0.56–0.85). Each more recent year of starting ART was associated with an increased risk of viral rebound (aHR = 1.29 [95% CI: 1.26–1.33]).

Figure 3B shows cumulative probabilities of confirmed virological failure, which were also lower in older adults. After three years of ART, the probability was 10.7% (95% CI: 7.6%–14.9%) in older adults and 15.7% (95% CI: 14.8%–16.6%) in younger adults (*P* = 0.003). In adjusted analyses, older adults had a 36% lower risk of virological failure, aHR = 0.64 (95% CI: 0.47–0.89). Covariates included in the model were baseline CD4 cell count, aHR = 0.99 (95% CI: 0.99–1.00), and nevirapine (compared to efavirenz) as the choice of initial regimen non-nucleoside reverse transcriptase inhibitor, aHR = 1.35 (95% CI: 1.22–1.52).

The proportion of patients who switched to second-line ART during the study period was a third lower in older adults (0.88% vs. 1.81% in older and younger adults, respectively); adjusted risk ratio (aRR) = 0.66 (95% CI: 0.43–1.00). Patients who started nevirapine had substantially increased switch to second-line ART, aRR = 3.35 (95% CI: 2.96–3.80).

After three years of ART, the median CD4 cell counts amongst older and younger adults were 377 cells/µL (IQR: 272–494 cells/µL) and 411 cells/µL (IQR: 279–564 cells/µL), respectively (P = 0.07; n = 4358). Amongst patients with continuous viral suppression, the difference between median CD4 cell counts after three years of ART amongst older and younger adults was greater, being 376.5 cells/µL (IQR: 279.5–474 cells/µL) and 424 (IQR: 300–576 cells/µL), respectively (p = 0.0022; n = 3024). The proportion of patients after three years with suppressed viral loads having CD4 cell counts <200 cells/µL (immune-virological discordant response) in older and younger adults were 8.7% and 5.9%, respectively (P = 0.162; n = 3169).

**Figure 4:**
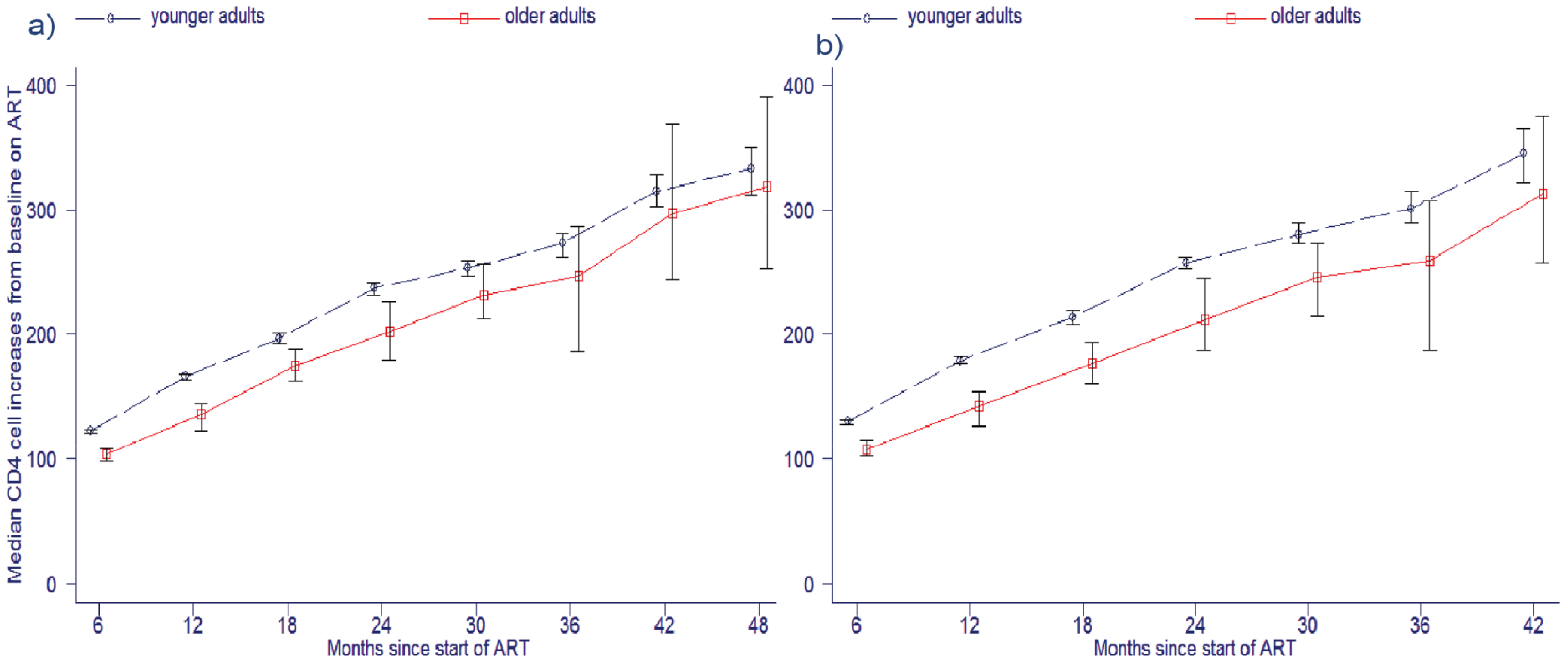
Median CD4 cell increases from baseline on antiretroviral treatment (ART) for older and younger adults in a) full cohort and b) limited to patients with continuous viral suppression. Error bars are 95% confidence intervals.

Figure 4 shows rates of CD4 cell increases from baseline in the whole cohort (Figure 4A) and in a subset of 28,816 patients with continuous viral suppression (Figure 4B). CD4 cell recovery was slower in older adults, with an adjusted modelled difference of 17.1 cells/6 months of ART (95% CI: 10.3 to 24.0) lower rate of increase in older adults in the whole cohort (n = 164,998 observations). In the subset with continuous viral suppression, the difference between older and younger adults was more marked, the adjusted modelled difference being 25.0 cells/6 months of ART (95% CI: 17.3–33.2) lower rate of increase in older adults. Covariates included in the model were: male gender, −41.7 cells/6 months of ART (95% CI: −45.3 to −38.0); time since starting ART, +40.4 cells/6 months of ART (95% CI: 39.7–41.1); year of starting ART, +7.9 cells/6 months of ART (95% CI: 6.8 to 8.9); and baseline CD4 cell count, −0.27 cells/6 months of ART (95% CI: −0.29 to −0.25).

## Discussion

This study of a very large multicentre cohort of patients in the South African public sector ART programme with up to five years of follow-up has shown that, compared to younger adults, older adults had better virological outcomes, reduced LTFU and a lower proportion who switched to second-line ART. Despite this, older adults had increased mortality and poorer immunological recovery than younger adults.

Few studies have reported virological outcomes specifically amongst older adults on ART in sub-Saharan Africa, particularly beyond 6–12 months of treatment. Older adults in this cohort had better virological suppression and reduced incidences of both viral rebound and confirmed virological failure. This likely explains the lower proportion of older adults that were switched to second-line ART. Virological outcomes have similarly been found to be better in older adults in developed countries [8], [10], [13], which may be as a result of improved adherence in older age [5], [7], [38].

The finding that LTFU amongst older adults is somewhat reduced is noteworthy, as LTFU is the major contributor to program losses in sub-Saharan Africa [39] and considering that clinic attendance is not diminished in older age despite comorbidities and cognitive decline that may occur with aging. Younger adults are also more likely to be mobile and migrate for work, which may contribute to the higher risk of LTFU in this group.

When mortality is high, as in sub-Saharan African ART programs, standard Kaplan-Meier analyses that ignore the competing risk of death substantially overestimate the cumulative incidence of LTFU [33]. However, competing-risks methods as used in this analysis avoid this bias [33]. Although patients who fulfilled the definition of LTFU may later have reengaged in treatment programs, we used a definition of ≥6 months absence from the last clinic visit which is shown to be associated with the lowest misclassification rate of active patients vs. patients LTFU [29].

Mortality in older adults receiving ART in sub-Saharan Africa has not been consistently higher than that of younger adults in two previous studies [17], [40]. However, the increased mortality amongst older adults in this study concurs with results from developed countries and a collaborative analysis from sub-Saharan Africa [18], [41]. Similar to the sub-Saharan African study, older age in this study was more weakly associated with early ART mortality than was the case with mortality over longer durations of ART [18]. Early mortality in sub-Saharan African settings is considerably higher than in developed countries, and is primarily due to more advanced HIV disease stage, greater levels of immunodeficiency and higher proportions who have co-infections when commencing ART than in developed countries, and not due to differences in patient age [42], [43].

Age-specific mortality rates during the later ART period amongst older adults in this study were between 37% to 62% higher than amongst older adults in the general South African population [37]. The reasons for increased later mortality on ART are complex and multi-factorial, and include, in addition to natural biological aging, the increased risk of HIV-associated non-AIDS conditions, the consequences of chronic inflammation, HIV-related accelerated biological aging, malignancies, and antiretroviral-specific drug toxicities [6], [8], [9], [14]. The poorer immune recovery observed in older patients also probably plays a role, resulting in increased susceptibility to opportunistic infections while on ART.

In HIV-negative populations, CD4 cell counts have been found to gradually increase until age 64 years, and then decline slightly with increasing age [44], [45]. In contrast, amongst HIV-positive people not receiving ART, increasing age (from 40 years and older) is associated with a more rapid decline in CD4 cell counts [46]. As in this study, older adults in developed countries have a slower CD4 cell count recovery after starting ART [13], [15]. This poorer immune recovery is thought to relate to, amongst others, an earlier onset of immunosenescence in the presence of HIV infection [4].

In developed countries, older adults commence ART at a later stage in the course of infection than younger people, primarily due to late HIV diagnosis [15]. In contrast, in this study, lower proportions of older adults started ART with extreme immunodeficiency or with advanced clinical stage disease than younger adults, which is similar to results from other sub-Saharan African cohorts [19], [40]. The reasons behind these findings are not easily explained, as older adults in sub-Saharan Africa are less likely to be tested for HIV, have lower levels of HIV awareness and display lower levels of health-seeking behaviour [47], [48].

The higher proportion of men amongst older patients reflects age-specific HIV prevalence trends in South Africa, as HIV prevalence in older men is estimated to be at least double that of older women [49], due to patterns of intergenerational sex between older men and younger women [50]. In addition, it may be related to gender differences in health-seeking behaviour as men tend to delay their presentation for treatment until later stages of infection [51], [52].

The proportion of adults falling in the older category was two-thirds higher at rural facilities than urban facilities, which is likely related to higher rural-urban migration amongst younger adults [53], and back-migration to rural areas by older adults. This has implications for rural healthcare services, where personnel may be undertrained and resources are limited to a greater degree [54], where greater numbers of older adults need to be managed whose care is likely to be more complex due to co-morbidities.

The strengths of this study include that data from a large number of patients from many sites were included, which allowed precise estimation of effect measures. The sites included were routine treatment facilities within the national public sector ART programme, the types of facility at which the vast majority of patients receive ART in sub-Saharan Africa, thereby reflecting the situation at an operational level and making the findings generalizable.

The limitations of the study include that due to the routine nature of the data, mortality amongst those LTFU was not ascertained, and causes of death were not available. Apart from mortality, clinical implications of poorer CD4 cell recovery amongst older adults were not measured, such as incident tuberculosis or AIDS-defining illnesses, as these data were not collected. Missing CD4 cell count and viral load results were prevalent; however, missing data values in routine ART programmes in sub-Saharan Africa are frequent [39], [55], [56]. Adherence determination data were not collected as they do not form part of the routine data captured for public-sector ART patients in South Africa. Socio-economic factors may be associated with mortality; however, socio-economic data were not routinely available.

In conclusion, routine HIV care and treatment services in sub-Saharan Africa need to anticipate increased numbers of older patients on ART (particularly in under-resourced rural areas) and prepare healthcare services for this vulnerable group as the population living with HIV ages. Increased mortality and poorer immune recovery amongst older adults provide rationale for further research to understand the mechanisms for this. Consideration should be given for the development of age-specific ART guidelines and eligibility criteria in resource-poor settings in order to improve longer term outcomes [19]: These may include (but not be limited to) the provision that older adults can initiate ART at higher CD4 cell counts due to their slower immunological reconstitution; enhanced screening and clinical vigilance for opportunistic infections amongst older adults; increased renal and hepatic function monitoring and more intensive considerations regarding polypharmacy in older adults; and the incorporation of chronic disease management strategies together with HIV management. Additionally, co-morbidities amongst older patients on ART in sub-Saharan Africa, which may contribute to the higher mortality we observed, are a research priority.
